# Extracellular vesicles remodel tumor environment for cancer immunotherapy

**DOI:** 10.1186/s12943-023-01898-5

**Published:** 2023-12-13

**Authors:** Ming Yue, Shengyun Hu, Haifeng Sun, Baojing Tuo, Bin Jia, Chen Chen, Wenkang Wang, Jinbo Liu, Yang Liu, Zhenqiang Sun, Junhong Hu

**Affiliations:** 1 Department of Colorectal Surgery, The First Affiliated Hospital of Zhengzhou University, Zhengzhou, 450052 Henan China; 2 Henan Institute of Interconnected Intelligent Health Management, The First Affiliated Hospital of Zhengzhou University, Zhengzhou, 450052 Henan China; 3 Department of Oncology, The First Affiliated Hospital of Zhengzhou University, Zhengzhou, 450052 Henan China; 4 Department of Breast Surgery, The First Affiliated Hospital of Zhengzhou University, Zhengzhou, 450052 Henan China; 5 Department of Radiotherapy, Henan Cancer Hospital, Affiliated Cancer Hospital of Zhengzhou University, Zhengzhou, 450001 China

**Keywords:** Extracellular vesicles, Tumor microenvironment, Cancer immunotherapy, PD-1, Non-coding RNA, Lymph node microenvironment, Engineered EVs

## Abstract

Tumor immunotherapy has transformed neoplastic disease management, yet low response rates and immune complications persist as major challenges. Extracellular vesicles including exosomes have emerged as therapeutic agents actively involved in a diverse range of pathological conditions. Mounting evidence suggests that alterations in the quantity and composition of extracellular vesicles (EVs) contribute to the remodeling of the immune-suppressive tumor microenvironment (TME), thereby influencing the efficacy of immunotherapy. This revelation has sparked clinical interest in utilizing EVs for immune sensitization. In this perspective article, we present a comprehensive overview of the origins, generation, and interplay among various components of EVs within the TME. Furthermore, we discuss the pivotal role of EVs in reshaping the TME during tumorigenesis and their specific cargo, such as PD-1 and non-coding RNA, which influence the phenotypes of critical immune cells within the TME. Additionally, we summarize the applications of EVs in different anti-tumor therapies, the latest advancements in engineering EVs for cancer immunotherapy, and the challenges encountered in clinical translation. In light of these findings, we advocate for a broader understanding of the impact of EVs on the TME, as this will unveil overlooked therapeutic vulnerabilities and potentially enhance the efficacy of existing cancer immunotherapies.

**Supplementary Information:**

The online version contains supplementary material available at 10.1186/s12943-023-01898-5.

## Introduction

Immunotherapy was developed based on tumor escape, by manipulating the body's immune system to reactivate the antitumor effect [1]. Tumor immunotherapies include immune checkpoint inhibitors (ICI), pericyte therapies, lysing viruses, and cancer vaccines. Immunotherapy, represented by immune checkpoint blockade, has transformed the treatment of many solid and hematologic malignancies [2–4]. However, only 10–30% of solid tumors are treated clinically, and the treatment sensitivity is low. TME is a complex environment for tumor survival. Tumor cells and their microenvironment interact and co-evolve to promote tumor generation, development and metastasis. During the process of tumor development, the TME dynamically changes, generating an immunosuppressive microenvironment that leads to tumor immune escape. Similarly, the heterogeneity of TME allows for individual differences in tumors, which may be related to individual differences in tumor immunotherapy [5].

Extracellular vesicles (EVs) released by various cell types and play an important role in intercellular communication. As an important component of tumor-host interactions, EVs are increasingly recognized as a key molecular entity in constructing the immunosuppressive microenvironment in TME. Tumor cells actively release EVs into the surrounding microenvironment, and these vesicles have pleiotropic abilities in regulating tumor growth progression, neovascularization, immune escape, and promoting tumor invasion and metastasis [6, 7]. Thus, EVs regulate intercellular communication not only between cancer cells, but also between cells in TME [8].

Tumor-induced immune cell exhaustion represents a pervasive stratagem employed to elude vigilant immune surveillance [9]. Notably, the wellspring of malignancy expels a copious profusion of these EVs, adorned with the effigy of programmed death-ligand (PD-L1) upon their lipid bilayer. The advent of anti-PD1 antibodies, employed to obstruct the PD1/PD-L1 axis and forestall immune cell exhaustion, has indeed wrought a profound transformation in the therapeutic landscape for advanced malignancies [10, 11]. However, notwithstanding the substantial triumphs attributed to this therapeutic paradigm, a litany of both preclinical and clinical investigations has elucidated the emergence of resistance mechanisms, whether innate or acquired, to anti-PD1 therapy. PD-L1, recognized interchangeably as CD274 or B7-H1, manifests as a transmembrane protein of considerable complexity, spanning 290 amino acids and encoded by the CD274 gene. This multifaceted protein encompasses immunoglobulin V-like and C-like extracellular domains of intricate architecture [12]. Emerging evidence has suggested that tumor-derived EVs (TEVs) carry biologically active PD-L1 on their surface, leading to the suppression of immune responses [13]. It is worth noting, however, that the corpus of literature referenced herein exclusively scrutinizes tumor-derived extracellular vesicular PD-L1, and while the significance of PD-L1 expression in bone marrow cells in the context of immunosuppression is undeniable, no investigations pertaining to extracellular vesicular PD-L1 emanating from bone marrow cells have graced the scientific discourse thus far.

Non-coding RNAs (ncRNAs), a category of ribonucleic acids devoid of protein-coding potential [14]. Nonetheless, in recent times, a subset of these ncRNAs has been discovered to possess the ability to encode peptides and proteins [15]. This subset encompasses distinguished entities such as microRNA (miRNA), long-stranded non-coding RNA (lncRNA), and circular RNA (circRNA), all of which exhibit well-defined functional roles. Furthermore, there exist enigmatic RNAs whose functional significance remains elusive. It is worth noting that these entities are transcribed from the genome, yet they do not undergo translation into proteins [16–18]. Also, EVs shelters ncRNAs capable of traversing to neighboring or distant cells. There, they orchestrate gene expression by modulating the levels of transcription factors or miRNAs. In doing so, they intricately regulate a myriad of cellular processes, encompassing but not confined to cell proliferation, differentiation, and programmed cell death [19, 20].

This review aims to provide an extensive examination of the diverse effects of EVs from various sources on the distinct components of the TME, with particular emphasis on the role of PD-L1 and non-coding RNA from EVs in TME remodelling and their effects across different cancer types. Furthermore, we also discuss the remodeling of the lymph node microenvironment by EVs and the potential of engineering EVs to enhance tumour immunotherapy. Taken together, these findings highlight the promising potential of EVs-mediated remodeling of the TME as a powerful strategy for future cancer immunotherapy applications.

### EVs and TME

EVs constitute a diverse array of membranous structures originating from various cellular sources, encompassing exosomes and microvesicles. They arise either from the endosomal system or shed from the plasma membrane, giving rise to this heterogeneous population [21, 22]. These vesicles serve as a crucial means of intercellular communication, facilitating the transfer of cellular components, such as lipids, proteins, and nucleic acids between cells [23–25]. They are characterized by high bioavailability, biostability, biocompatibility, and cargo loading capacity, which render them attractive candidates for therapeutic interventions. The unique features and functions of these vesicles are highly influenced by the cell type of origin, cellular state, and the surrounding microenvironment. These factors collectively contribute to the heterogeneous nature of EVs and emphasize the need for a comprehensive understanding of their biology to exploit their full potential in cancer therapy [26, 27]. EVs may be involved in various physiological and pathological processes, including cell cycle, apoptosis, angiogenesis, thrombosis, immune inflammation, fibrosis, and tumor development [28–35]. EVs deliver information to recipient cells through three different pathways: (1) direct contact of proteins on the EVs membrane with proteins on the recipient cell membrane, which then triggers an intracellular signaling cascade [36]; (2) fusion of the EVs membrane with the recipient cell membrane and release of its contents into the recipient cell [37]; (3) direct phagocytosis of the EVs by the target cell and internalization of the EVs into its components [38]. Based on the physical, chemical, and biological properties of EVs, different methods have been developed to isolate and purify EVs, including differential centrifugation, density gradient centrifugation, magnetic bead sorting, multimer-dependent, microfluidic, and EVs extraction kits with immunological methods [39] (Table 1). The most widely accepted method is differential centrifugation.

**Table 1:** Methods for isolating and characterizing EVs

According to a survey conducted by the International Society for Extracellular Vesicles (ISEV), the current methods commonly employed for the isolation and purification of EVs include differential centrifugation, density gradient centrifugation, filtration, size-exclusion chromatography, and immunological approaches, with differential centrifugation comprising 81% of the total [40]. It is advisable to employ a combination of isolation methods to clearly segregate subpopulations of vesicles based on their size, density, or composition, thus enhancing credibility. Differential centrifugation initially employs low-speed centrifugation to separate cell debris and larger particles from the matrix, followed by high-speed centrifugation to precipitate EVs from cellular metabolites and other substances [41]. While this method is widely used, it comes with certain drawbacks, such as time-consuming procedures, the requirement of substantial initial sample volumes, the need for expensive equipment, and relatively low EVs extraction purity [42, 43]. Recently, microfluidics, as an emerging method for EVs isolation, has been gaining prominence. Microfluidic technology offers numerous advantages, including cost-effectiveness and high sensitivity. The objective of this technology is to capture, filter, separate, and purify exosomes based on their physical and chemical properties [44, 45]. This emerging approach provides researchers with additional choices Methods commonly used to identify and assess EVs primarily involve the characterization of their physicochemical properties, specifically particle size, concentration, morphology, and cargo-carrying capabilities. Physical analyses encompass techniques such as nanoparticle tracking analysis (NTA), dynamic light scattering (DLS), electron microscopy, and tunable resistive pulse sensing (tRPS). Both NTA and DLS operate on similar principles, relying on the detection of scattered light signals generated by particle Brownian motion within a specific range. Subsequently, these signals are captured using microscopy or other collection devices, and the Stokes–Einstein equation is then employed to calculate particle size and concentration [46, 47]. Biochemical analyses are typically conducted through methods such as flow cytometry, immunoblotting, or proteomic analysis, providing data regarding the components found within the isolated vesicles. Flow cytometry, for instance, is generally considered suitable for counting EVs ranging from 300 to 500 nm in size [48]. However, analysis of smaller-sized EVs can be accomplished using antibody-coated magnetic beads. Immunoblotting, on the other hand, identifies EVs by the specific binding of antigen and antibody. It is imperative to note that, as outlined by ISEV, in order to validate the EV properties and purity of an EV preparation, it is necessary to analyze transmembrane or GPI-anchored proteins located in the outer membrane of prokaryotic cells and cytoplasmic or periplasmic proteins to demonstrate the presence of EVs and evaluate their purity from common contaminants [49]	

EVs play a key role in cellular communication between tumor cells and stromal cells in local and distant microenvironments. The TME constitutes a complex and heterogeneous milieu comprising various cellular and non-cellular components, such as blood vessels, immune cells, fibroblasts, extracellular matrix, metabolic waste products(e.g., lactic acid), and signaling molecules(e.g., chemokines, cytokines, growth factors, etc.) [50]. Low oxygen levels, high lactate levels, extracellular acidosis, and poor nutrient composition are prominent features of TME [51–53]. In *vivo* and ex *vivo* studies of the interaction of EVs with TME varied (Table 2). TME plays an integral role in cancer biology and is involved in tumorigenesis, progression, and response to therapy. TME harbors a plethora of immunosuppressive molecules and immunosuppressive cells, including regulatory T cells (Tregs), M2-type macrophages, TIM-3, IL-2, which collectively promote immune evasion and cancer progression. The intricate interplay between malignant, endothelial, stromal, and immune cells within the TME regulates its homeostasis and evolution. On the contrary, EVs represent pivotal factors that establish communication channels among these distinct cellular compartments.

**Table 2:** Discussion of in vitro and in vivo methods for studying the role of EVs in TME

For investigating the intricate interplay between EVs and the TME cells, whether in vitro or in vivo, the primary approach revolves around employing fluorescence labeling of EVs through liposomal bilayers or using fluorescently tagged cargos embedded within these vesicles. Typically, EVs are labeled using lipophilic dyes such as PKH67 (green fluorescence)/PKH26 (red fluorescence) and Di-series dyes. Alternatively, specific proteins like CD63 and the green fluorescent protein (GFP) expression elements within exosomes can be engineered into plasmids and subsequently packaged into lentiviruses. These lentiviruses are then used to infect cells, resulting in the secretion of exosomes adorned with green fluorescence [54]. Depending on the objectives, models, and TME analyses, EVs can be administered through subcutaneous injection, intravenous injection, or intraperitoneal injection. After a certain duration, live animal imaging systems are employed for in vivo tracking, or the experimental animals are euthanized, and target tissues are collected for imaging observations, thereby assessing whether EVs have been taken up by the target cells within the TME. In contrast, in vitro experiments typically involve co-culturing these exosomes with the cells under study for a certain period. Fluorescence signals are observed using imaging instruments like laser confocal microscopes The functional analysis of the intricate interplay between EVs and the TME is contingent upon the specific research questions at hand. In both in vivo and in vitro experiments, methods such as immuno-electron microscopy and enzyme-linked immunosorbent assay (ELISA) can be employed to investigate whether extracellular structures bind to target cells [55], particularly when studying the membrane surface proteins of EVs. For studying the proteins and lipids contained within exosomes, proteomic and lipidomic analyses are conducted to trace the protein and lipid components, their types, and their interactions with other biomolecules within target cells. Immunoprecipitation methods can also be used for proteins [55]. In the case of non-coding RNAs encapsulated within EVs, RNA immunoprecipitation is an applicable technique. The distinction between in vitro and in vivo research lies primarily in the analysis of target cells or tissues. In vitro experiments involve co-culturing EVs with target cells, and upon confirming the internalization of EVs by target cells, functional changes such as cytokine expression, cell proliferation, migration and invasion, apoptosis, and luminal formation experiments are observed. In vivo experiments, on the other hand, encompass assessing tumor volume, mass, or conducting single-cell RNA sequencing analysis to elucidate cellular functional changes within animal tissues	

### Modulating different cells in TME with EVs to influence immunotherapy

Tumor tissues are like alien life forms that do whatever they can to survive in the organism. Tumor cells recruit normal tissue cells and induce conditions that produce favorable tumor growth. The effective amplification and metastasis of cancer cannot be separated from the immune escape ability of cancer cell or treatment-mediated immune surveillance [56–59]. EVs derived from tumor cells are crucial targets in the intricate network of tumor immunity [60]. These EVs have the ability to dampen immune function, foster the differentiation of regulatory T cells and tumor-associated macrophages, and even substitute tumor cells with immune cell assaults to bolster tumor cell immune tolerance and evade immune surveillance [61–63]. Cancer cell derived EVs help cancer cells grow, metastasize and become resistant to drug therapy [64, 65]. Herein, we elucidate pivotal factors in the current landscape of EVs reshaping the TME and their impact on immunotherapy (Table S1).

#### CD8 T cell

**Fig. 1:**
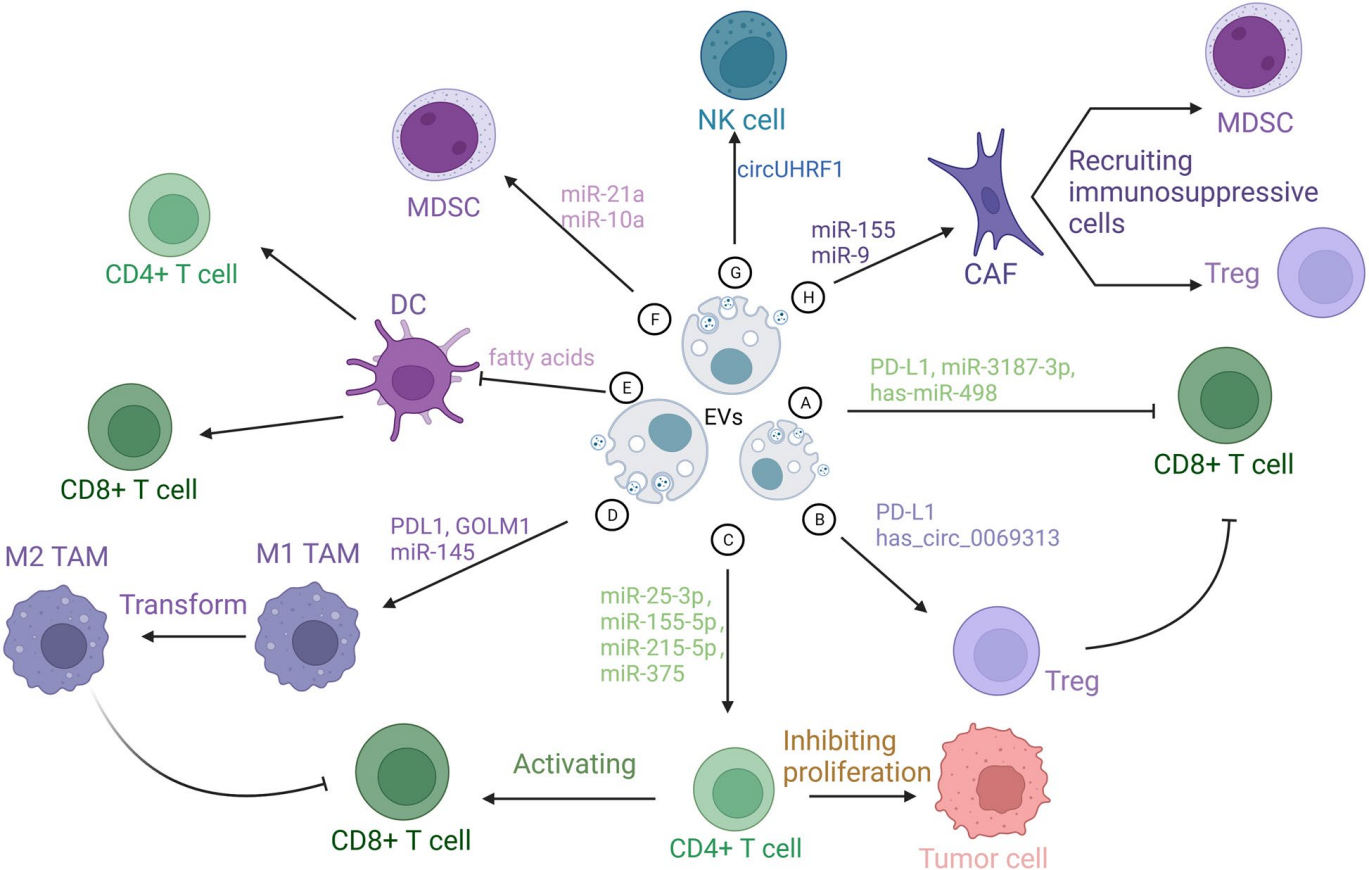
EVs regulating different cells in TME.** A** TEV induces CD8 T-cell depletion. **B** TEV activates Treg and thus suppresses CD8 T cell function. **C** CD4-derived EVs activate CD8 T cells and inhibit the cell cycle of tumour cells. **D** TEV induces a shift from M2 to M1 type macrophages. **E** Endocytosis of TEV-containing fatty acids leads to impaired DC cell function and reduced ability of antigen presentation to activate T cells. **F** TEV activates MDSCs. **G** TEV induces NK cell depletion. **H** TEV activates CAF, thereby recruiting MDSCs and Treg in TME

EVs assume a pivotal role in sculpting the intricate tapestry of the TME. These minute vesicles serve as discreet couriers, ferrying an array of immunosuppressive molecules that wield profound influence over the delicate balance of tumor immunity [66]. PD-L1, a linchpin molecule in the domain of immune regulation, serves as the harbinger of T cell exhaustion [67]. The indispensable role of CD8 T cells in the realm of tumor immunity should not be underestimated. Nevertheless, tumor tissues frequently pursue their own agenda by instigating the depletion of these CD8 T cells. It has been empirically validated that the interplay between PD-L1 adorning the tumor's surface and PD-1 residing on CD8 T cells, through their extracellular structural domain, culminates in the quelling of T-cell functionality [68–70]. Tumorous cells possess the capacity to liberate EVs, which enact direct influence upon CD8 T cells enmeshed within the intricate TME. Incipient investigations augur that extracellular vesicular PD-L1 may serve as a stratagem to counteract immune pressures during the effector stage, with a selective affinity for PD-1 + CD8 + T cells [55]. The PD-L1 adorning the surface of cancer cell EVs engages with PD-1 receptors on CD8 + T cells, resulting in the impediment of CD8 + T cell proliferation and activation [71, 72] (Fig. 1A). Notably, impeding the exodus of these EVs was accompanied by a marked deceleration in tumor progression [72]. EVs, derived from tumor cells, have emerged as pivotal arbiters in orchestrating the TME through the dissemination of their cargo. Upon internalization of TEVs, both the quantity and functionality of CD8 + T cells exhibit diminishment [73]. Furthermore, these EVs wield the capacity to modulate the expression of cell surface moieties within the TME. The bestowal of EVs upon CD8 + T cells culminates in their enervation and the concomitant suppression of tumor immunity, as evinced by a substantial reduction in the expression of TNF-α, IFN-γ, granzyme-B, and perforin [74]. It is noteworthy that the ICAM-1 expressed on cloaking PD-L1 engages with LFA-1 on the surface of CD8 + T cells, a pivotal interaction for the binding of TEXs with T cells. Simultaneously, the secretion of IFN-γ by CD8 + T cells upregulates the expression of ICAM-1 [75].

**Fig. 2:**
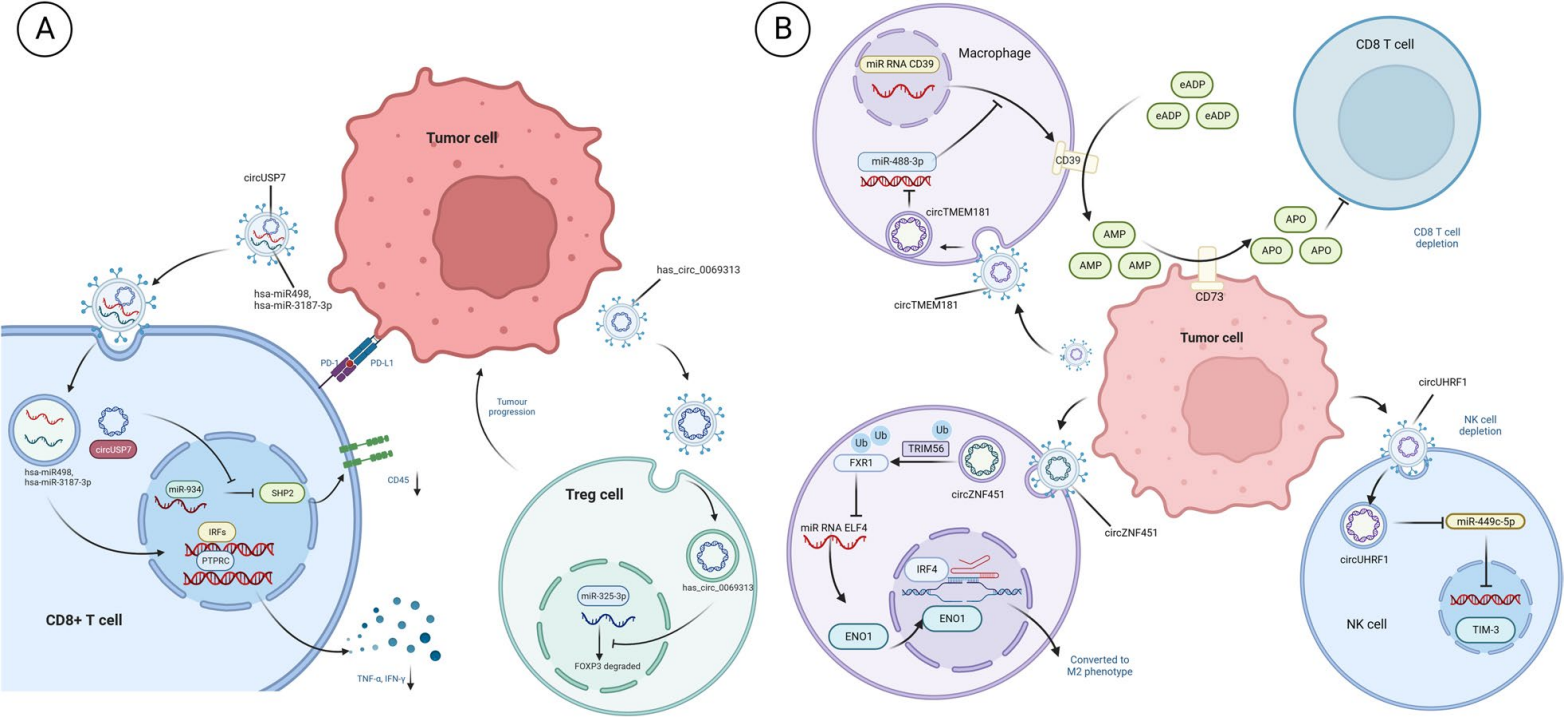
Mechanisms of remodelling TME by TEVs.** A** Mechanisms by which TEV affects T cells. TEV interferes with CD8 T cells and induces their depletion, while activating Treg, thereby affecting immunotherapy. **B** Mechanisms by which TEV affects macrophages and NK cells. TEV affects the expression of CD39 in macrophages and induces conversion to M2. TEV also induces depletion of NK cells, depriving them of their tumour-killing capacity

The impact of ncRNAs within EVs on immunotherapies in the TME is profound. These RNAs wield their influence by modulating T-cell activation and functionality, ultimately suppressing immune responses [30]. For instance, the transfer of EVs- derived circUSP7 from non-small cell lung cancer tissue to CD8 + T cells results in the suppression of miR-934 expression. This, in turn, upregulates downstream molecules of SHP2, impairing the function of CD8 + T cells. The consequence is a decrease in TNF-α and IFN-γ expression, leading to reduced CD8 + T cell counts and tumor immune evasion. Notably, in HuNSG mice endowed with a humanized immune system, high circUSP7 expression in NSCLC cells confers resistance to PD1 therapy [74]. Similarly, extracellular vesicular hsa-miR-498 originating from melanoma acts on the 3'UTR of TNF-α in T cells, resulting in downregulation of its expression. Likewise, hsa-miR-3187-3p targets the 3'UTR end of PTRRC, leading to transcriptional downregulation of its encoded CD45. These actions contribute to tumor immune evasion [73] (Fig. 2A). Given melanoma's pronounced metastatic propensity, researchers have delved into this domain and identified melanoma derived EVs that promote lymphatic vessel remodeling and expansion. These EVs also carry tumor antigens and induce CD8 + T cell apoptosis, thereby enhancing melanoma metastasis [76]. EVs derived from pancreatic cancer (PC) cells have been shown to activate the P38 MAPK signaling pathway, ultimately culminating in CD8 + T cell apoptosis. Unfortunately, the specific extracellular vesicular molecules responsible for this effect remain unclear [77]. These findings underscore the potential of extracellular vesicular ncRNAs as attractive therapeutic targets to enhance the efficacy of immunotherapies, albeit with the consequence of diminished T cell function and anti-tumor effects.

#### CD4 T cell

PD-L1 induces the generation of Tregs in a PD-1-dependent manner [78]. In head and neck squamous cell carcinoma (HNSCC), there is a significant positive correlation between the expression of FOXP3 mRNA and CD163 mRNA and TEV-PD-L1 features. Furthermore, it has been demonstrated that TEVs derived from PD-L1-rich HNSCC play a pivotal role in inducing Treg activation and differentiation [79]. Like other immune cells, the content of EVs can also upregulate PD-L1 expression in Tregs. Administration of EVs derived from oral squamous cell carcinoma (OSCC) results in an upregulation of PD-L1 expression in Tregs. Furthermore, upon uptake, these EVs carrying has_circ_0069313 facilitate Treg activation by impeding miR-325-3p-mediated Foxp3 degradation, thus promoting immune evasion [80] (Fig. 1B).

EVs derived from CD4 T cells, specifically carrying miR-25-3p, miR-155-5p, miR-215-5p, and miR-375, have been identified as key instigators of CD8 T cell-mediated anti-tumor responses. In a melanoma mouse model, these EVs effectively suppress tumor growth by activating CD8 T cells [81] ( Fig. 1C). Additionally, CD4 + T cells secrete IL-2, a crucial factor for initiating cytotoxic T lymphocyte (CTL) responses through CD8 + T cells [82]. It's worth noting that EVs derived from IL-2-stimulated CD4 T cells induce a more robust anti-tumor response in CD8 T cells when compared to those from unstimulated CD4 T cells [81]. This phenomenon represents a mechanism by which the immune system combats the immunosuppressive microenvironment in cancer.

#### Vδ2 T cells

Vδ2 T cells, due to their MHC-independent direct anti-tumor activity and their potential in adoptive cell therapy, hold promise as candidates for cancer immunotherapy [83–86]. Vδ2-T-Exos contain death-inducing ligands (FasL and TRAIL) as well as immune-stimulatory molecules (CD80, CD86, I and II class MHC). In immunodeficient and humanized mouse models, Vδ2-T-Exos effectively target EBV-related tumor cells and induce efficient killing of these cells through the FasL/Fas and TRAIL/DR5 pathways. This approach effectively controls EBV-related tumors, targets and efficiently eliminates EBV-related tumor cells through the FasL and TRAIL pathways and promotes the expansion of EBV antigen-specific CD4 and CD8 T cells [87].

#### Tumor-associated macrophage

In the cellular communication between tumor and CD8 + T cells, tumor-associated macrophage (TAM) acts as an "intermediate transduction station" to receive signals contained in EVs and deliver them to CD8 + T cells. The PD-L1 molecules exhibited on TAMs entangle with PD-1 receptors displayed on CD4 + and CD8 + effector T cells, instigating the curtailment of T-cell activity and instigating apoptosis through the inhibition of T Cell Receptor (TCR) signaling [88]. Nevertheless, the eradication of PD-L1-expressing TAM infiltration and the mitigation of CD8 + T cell suppression have been demonstrated to assume a pivotal role in enhancing the efficacy of anti-PD-L1 immunotherapy. GOLM1, through the upregulation of CSN5 expression, attenuates the ubiquitination of PD-L1, thereby enhancing the stability of PD-L1. Furthermore, GOLM1 facilitates the sorting of PD-L1 into EVs by restraining Rab27b in the trans-Golgi network region. TAMs internalize these EVs carrying PD-L1, resulting in an elevated expression of PD-L1 on TAMs compared to hepatocellular carcinoma (HCC) cells. This, in turn, induces the suppression of CD8 T cells [89]. EVs also translocate PD-L1 to other cell types in the TME, leading to immunosuppressive effects, such as monocytes、macrophages and CD4 T cells [55, 71, 72, 90]. Moreover, the utilization of HNSCC tumor cell supernatant containing TEVs precisely induce the differentiation of M0 macrophages into the M2 phenotype. Furthermore, the expression of TEV-PD-L1 characteristics is highly positively correlated with the M2 phenotype in the TME. Further investigation revealed that TEV-PD-L1 induces macrophages to differentiate into the M2 type [79]. Interestingly, extracellular vesicular components other than PD-L1 have been found to elevate PD-L1 expression in TAMs. Evidence indicates that HCC cells, under endoplasmic reticulum (ER) stress, release extracellular vesicular miR-23a-3p. This miRNA regulates PD-L1 via the PTEN-PI3K-AKT pathway. In vitro experiments confirmed that miR-23a-3p inhibits PTEN, leading to increased phosphorylated AKT and PD-L1 expression in macrophages [91].

As previously underscored, macrophages play a pivotal role in the interaction with tumor cells and CD8 + T cells. EVs originating from tumor tissues are internalized by macrophages and subsequently transmit signals to CD8 + T cells [92]. Recent investigations have unveiled that high expression of Tim-4 on lung-resident macrophages in lung cancer patients correlates with decreased levels of CD8 + T cells bearing characteristics associated with the tumor response, such as PD-1 and CD39 [93]. Adenosine suppresses immune cells, rendering them exhausted [94]. Recent studies have shown that TAMs receive EVs containing circTMEM181, leading to increased expression of MiR-488-3p, subsequently enhancing CD39 transcription. The combination of CD39 + TAMs and CD73 + tumor cells result in the degradation of extracellular ATP into adenosine monophosphate (APO), inducing CD8 + T cell exhaustion [95] (Fig. 2B). TAMs promote cancer cell proliferation and migration through extracellular vesicular signaling [96, 97]. Additionally, EVs foster an anti-inflammatory phenotype in TAMs, reshaping the immunosuppressive TME [98]. TAMs are recruited from normal macrophages by tumor tissues [99] and exhibit two distinct phenotypes: pro-inflammatory M1 and anti-inflammatory M2 [100]. EV-associated miR-145 induces polarization of macrophages towards an M2 phenotype [101] (Fig. 1D). Recent findings have demonstrated that extracellular vesicular circZNF451 derived from lung adenocarcinoma (LUAD) triggers an anti-inflammatory phenotype in macrophages, resulting in reduced cytotoxic CD8 + T cells. Transgenic mouse studies have shown that extracellular vesicular circZNF451 targeting macrophages elevates the levels of TRIM56, deubiquitinates the FXR1 protein, thereby enhancing the ELF4-IRF4 pathway, leading to macrophage differentiation into an M2 phenotype. Deletion of ELF4 in macrophages rescues the efficacy of immunotherapy [98] (Fig. 2B). EVs originating from PC cells stimulate macrophage polarization into the M2 phenotype [102–105]. This potent immunosuppressive milieu, coupled with the proliferation of immunosuppressive cells, may account for the limited success of immunotherapy in managing pancreatic cancer. However, recent research indicates that the M1/M2 classification of macrophages is not reliable, and CXCL9 and Spp1 are more persuasive markers for macrophage polarization [106]. This knowledge should be updated in future relevant studies.

Furthermore, TAMs also transmit EVs to tumor cells. In hypoxic glioblastoma, M2 macrophages transport EVs enriched with miR-501-3p to tumor cells, resulting in the downregulation of TGFBR3 expression in tumor cells. Ultimately, this process promotes tumor cell invasion and migration through the TGF-β signaling pathway, accelerating tumor progression [107]. EVs derived from TAMs are rich in miR-95 and are taken up by prostate cancer cells. In vitro and in *vivo* loss-of-function experiments suggest that miR-95 acts as a tumor initiator by promoting prostate cancer cell proliferation, invasion, and epithelial-mesenchymal transition by directly binding to its downstream target gene, JunB [108].

#### Dendritic cells

As the principal antigen-presenting cells, dendritic cells (DCs) wield undeniable influence in the realm of tumor immunotherapy. Transporting fatty acids, TEVs orchestrate a symphony of immune dysfunction within DCs, facilitating a ballet of immune evasion. Specifically, these fatty acids, in a manner contingent upon the peroxisome proliferator-activated receptor alpha (PPARα), incite an excess of lipid droplet biogenesis and augmented fatty acid oxidation (FAO), thereby shifting the metabolic course of DCs from glycolysis towards mitochondrial oxidative phosphorylation, ultimately propelling immune functional impediments in DCs [54] (Fig. 1E).

DCs derived EVs (DEX) are enriched in MHC-I molecules and are potent inducers of T-cell responses [109, 110]. Specifically, DEX generated from tumor peptide-pulsed DCs induce tumor-specific CTL response in *vivo*, which activates the TME and enhances anti-tumor effects. In animal models, treatment with EVs derived from DCs expressing the tumor-associated antigen afetoprotein (DEXAFP) improved the tumor immune microenvironment of primary tumors [111].

#### Myeloid-derived suppressor cells

While tumor progression, there is a notable increase in myeloid-derived suppressor cells (MDSCs), and TEVs play a pivotal inductive role in this process. In murine tumor models, miR-21a found within TDEs has the remarkable capacity to significantly promote the expansion and suppressive function of MDSCs by targeting Programmed Cell Death Protein 4 (PDCD4). This occurs through the activation of interleukin-6 (IL-6) autocrine signaling and the phosphorylation of the STAT3 signaling pathway, consequently expediting tumor growth [112] (Fig. 1F). Hypoxia promotes the secretion and upregulation of miR-10a and miR-21 in glioblastoma-derived extracellular vesicles (GDEs). Furthermore, hypoxia-induced glioblastoma-derived extracellular vesicles (H-GDEs) stimulate the activation and differentiation of MDSCs via miR-10a and miR-21, acting through the targeting of the Rora/IκBα/NF-κB and Pten/PI3K/AKT pathways [113].

#### Natural Killer cells

Natural Killer (NK) cells, owing to their potent antitumor responses, have emerged as promising candidates in cancer therapy [114, 115]. They discern tumor cells in an antigen-independent manner, endowing them with the ability to circumvent immune evasion mechanisms involving the downregulation of MHC-I expression [116, 117]. Within this context, TEVs containing circUHRF1 lead to the degradation of miR-449c-5p (Fig. 1G), subsequently diminishing the transcription of genes encoding downstream target TIM-3. This, in turn, suppresses the production of IFN-γ and TNF-α by NK cells. Furthermore, augmented intercellular communication between NK cells and HCC cells might bypass the upregulation of TIM-3, resulting in impaired NK cell functionality and a phenotypic state of exhaustion [118] (Fig. 2B).

In a select cohort of Non-Small Cell Lung Cancer (NSCLC) patients, NK cells and their extracellular vesicles, as well as circulating tumor cells (CTCs), were isolated. Interestingly, NSCLC patients exhibited a substantial abundance of NK cells and NK-derived extracellular vesicles in comparison to healthy donors. Furthermore, a negative correlation was observed between CTC numbers and NK cells, while a positive correlation existed between CTC numbers and NK-derived extracellular vesicles. This intricate relationship may stem from increased CTC quantities subjecting more circulating NK cells to stress, compounded by the immunosuppressive microenvironment, potentially inducing active release of extracellular vesicles by NK cells [119].

#### Cancer-associated fibroblasts

As a predominant component of TME matrix, EVs originating from cancer-associated fibroblasts (CAFs) intricately modulate cancer progression through mechanisms such as facilitating cancer cell invasion and metastasis, as well as promoting immune evasion [120–123]. Research on myofibroblasts in hepatic stellate cells (HSC) suggests that CAFs could serve as pivotal sources of PD-L1 in various cancer types. Moreover, HSC PD-L1 modulate tumor growth independently of PD-L1/PD-1-mediated immune suppression by regulating the release of paracrine factors [124]. Both CAF-EVs and normal fibroblasts-EVs contain PD-L1 protein and mRNA. These PD-L1-containing EVs can be internalized by tumor cells. Concurrently, it has been observed that CAFs-Exos upregulate PD-L1 on the surface of lung cancer cells, thereby inhibiting the peripheral blood mononuclear cells' (PBMC) ability to induce the killing of lung cancer cells [125]. Similarly, in breast cancer, CAF-Exos exhibit analogous functions. These EVs originating from CAFs significantly impede immune cell function in *vivo* and promote the expression of PD-L1 in breast cancer cells [124].

Tumor cells also employ EVs to activate CAFs and influence T cell functionality. Research has elucidated that within TEVs, the presence of miR-155 serves as an inducer, orchestrating the genesis of CAFs [126]. Exposed to TEVs, the invasive capacity of fibroblasts has markedly intensified, surpassing its previous magnitude by approximately 2.5-fold. Additional experimental evidence suggests that normal stromal fibroblasts undergo phenotypic and functional alterations upon contact with EVs, transforming into CAFs [127]. Moreover, in triple-negative breast cancer, miR-9 orchestrates a CAF-like phenotype in tumor cells via EV-mediated pathways [128] (Fig. 1H). CAFs foster tumor growth by establishing an immunosuppressive microenvironment that attracts MDSCs and Tregs, further dampening T cell functionality [129–131]. Nevertheless, it remains unclear whether this newly discovered mechanism impacted immunotherapy through extracellular vesicular signaling.

#### Mesenchymal stem cells

Mesenchymal stem cells (MSCs) have garnered considerable interest in the realm of cancer therapy due to their low immunogenicity and ease of isolation and cultivation from adult tissues [132]. Extracellular vesicles derived from CD90 low adipose-derived mesenchymal stem cells (ADSC-EVs) exhibit a remarkable capacity to impede tumor growth in murine models. This phenomenon is intricately linked to the reduction in tumor cell proliferation and migration mediated by ADSC-EVs, accompanied by enhanced tumor cell apoptosis. In *vivo* experiments underscore the significant deceleration of tumor growth when employing miR-16-5p-mimicking loaded CD90 low ADSC-EVs [133]. This implies the potential applicability of extracellular vesicles produced by mesenchymal stem cells in immunotherapy. However, harvesting a sufficient yield of endogenous EVs from MSCs for clinical and research purposes has proven to be a formidable challenge. Fortunately, recent research has unveiled that treatment with cell-relaxant B can augment vesicular membrane production [134]. Employing cell-relaxant B treatment on a specific line of human adipose tissue-derived mesenchymal stem cells (hADSC), which overexpress TRAIL, PTEN, and IFN-β1, led to the isolation of artificial vesicles. These artificial vesicles not only activate human immune cells, such as CD8 T cells, ex *vivo*, but also induce apoptosis in cancer cells [135].

Interestingly, EVs derived from MSCs also possess immunosuppressive properties, as exemplified by miR-222 sourced from MSC-EVs. Upon transcriptional regulation of ATF3, miR-222 activates the AKT pathway, thereby promoting the development of colorectal cancer (CRC), enhancing tumor growth, and facilitating in *vivo* immune evasion [136]. This peculiarity may be associated with the heterogeneous nature of MSC subpopulations.

#### Neurons

In tumors, those endowed with greater neural dominance exhibit heightened invasiveness [137]. As cancer progresses, neural fibers sprout and infiltrate the tumor microenvironment, and the density of these nerves within solid tumors correlates with poorer prognosis [138]. In vitro experiments have elucidated that EVs released by HNSCCs can indeed promote neural outgrowth, a phenomenon highly associated with HPV infection [139]. Given the pivotal link between HPV infection and cervical cancer, researchers have extended their observations to the realm of cervical malignancies. During cervical cancer progression, a discernible shift in neural innervation within the cervix is noted. This is exemplified by a marked reduction in the expression of β-III tubulin coinciding with a pronounced increase in TRPV1 expression, all mediated by tumor-dependent EVs [140]. The origin and mechanisms underlying the emergence of these newly formed adrenergic nerve fibers that bolster tumor growth have long been shrouded in mystery. Recent investigations have shed light on this mystery in the context of TP53-deficient oral cavity squamous cell carcinoma (OCSCC), which are notably enriched with adrenergic nerve fibers. In TP53-deficient tumors, the content of miR-34a within secreted EVs undergoes a significant reduction. Contrasting this against a control group, these EVs promote neo-adrenergic cancer-associated neurogenesis. MiR-34a, derived from OCSCC, is shuttled via EVs to cancer-associated neurons, whereby it exerts a negative modulation on EV-derived axon growth signals, thereby fostering resistance. Conversely, TP53-deficient tumors release EVs with diminished miR-34a content, neutralizing the negative axon growth signals and bolstering the number of adrenergic nerve fibers, ultimately facilitating tumor progression [141]. Emerging research has also unveiled that Schwann cell-derived EVs possess the capacity to instigate macrophage polarization towards the M2 phenotype, thereby promoting remyelination and axonal regeneration following peripheral nerve injury [142]. Regrettably, whether such a scenario unfolds within the tumor microenvironment remains elusive.

In a word, recent investigations into the mechanisms underlying EV-mediated remodeling of the tumor microenvironment have largely centered around extracellular vesicular PD-L1 and ncRNAs. Notably, the role of the adenosine pathway in immunotherapy represents a fascinating and emerging area of research that expands our understanding of this field. However, the specific genes and signaling pathways that mediate these effects remain unclear and will require further investigation in future studies. Overall, tumor cells exploit EVs to suppress immune function and evade immune surveillance, thus hampering the efficacy of immunotherapeutic approaches. Conversely, normal cells, particularly immune cells, also release EVs to counteract this phenomenon [143, 144]. The constant battle between normal and tumor cells relies heavily on the interplay of extracellular vesicular signaling. Should the immune system triumph, it eradicates the tumor and re-establish the body's homeostasis. In contrast, if the tumor cells gain the upper hand, they hijack immune cells via microenvironmental cues.

### Hormonal effects in EVs remodelling TME

The interaction between tumor cells and the immune microenvironment relies upon intricate mechanisms, encompassing endocrine factors, pro-inflammatory states, and reciprocal influences among immune cells to regulate the TME [145]. Hormones are indispensable for the immune regulation within the body, and hormonal imbalances are, in part, factors contributing to tumor progression.

Estrogens are crucial drivers of estrogen receptor-positive (ER +) tumor progression. Estrogens increase the quantity of EVs in ER + breast cancer cells. ER + cell lines demonstrated a significant enrichment of Evs carrying either let-7a-5p, let-7c-5p, or let-7d-5p after treatment with 17β-estradiol, an effect not observed in ER-negative cells [146]. Interestingly, these 17β-estradiol-stimulated Evs subsequently attenuated the estrogen effect. Metastasis-associated protein 1 (MTA1) has been reported to prevent estrogen receptor-mediated transcription by interacting with histone deacetylases and nucleosome remodeling complexes [147]. MTA1 has also been identified in breast cancer-derived Evs, and it was confirmed to dampen estrogen signaling, as observed after stimulating breast cancer cells with 17β-estradiol [148]. In uterine corpus endometrial cancer, another estrogen-driven malignancy, low levels of miR-765 induced by estrogen triggered high proliferation, Epithelial–mesenchymal transition (EMT) processes, invasion, and poor prognosis via the activation of the PLP2-Notch signaling pathway. Interestingly, this effect could be partially suppressed by EVs carrying miR-765 released by CD8 T cells [149]. Estrogens offer a new perspective on the antitumor mechanisms of EVs in gynecological tumors, offering potential therapeutic avenues. Similar to gynecological tumors, prostate cancer presents issues related to androgen resistance. Unfortunately, the mechanisms of crosstalk between EVs and the immune microenvironment in this field remain unclear.

In non-hormone-driven tumors, hormones continue to play a significant role. Melatonin (MLT), known for its role in regulating circadian rhythms, has demonstrated antitumor activity in recent studies [150]. MLT modulates macrophage immune function and reduce cancer cell activity by regulating TEVs. EVs from gastric cancer(GC) cells treated with MLT promote the expression of TNF-α and CXCL10 in macrophages while suppressing IL-6 and MCP-1 expression, concurrently downregulating PD-L1 protein levels [151].

Surprisingly, hormones can also be contained within EVs. EVs from gallbladder cancer (GBC) harbor leptin and possess the capacity to upregulate leptin levels in TAMs. Elevated leptin levels activate the STAT3 signaling pathway, driving macrophage polarization towards the M2 phenotype, thereby promoting GBC cell invasion and migration [152].

In summary, hormones can modulate the reshaping of EVs in the tumor microenvironment, thereby influencing the immune system based on this principle. These mechanisms provide insights for future tumor immunotherapies.

## EVs inducing pre-metastatic niche formation

The pre-metastatic niche (PMN) is an intricately orchestrated microenvironment pre-established in distant tumor-free regions or organs, primed by the primary tumor, in anticipation of widespread metastasis [153]. Its hallmarks encompass immune suppression, inflammation, angiogenesis, vascular permeability, lymphangiogenesis, extracellular matrix (ECM) remodeling, and extracellular matrix (ECM) deposition [154, 155]. As an integral facet of cellular communication, extracellular vesicles assume a paramount role in shaping the pre-metastatic niche, a function of undeniable significance.

### The formation of Chronic inflammation and immunosuppressive microenvironment

The tumor microenvironment typically resides in a state of hypoxia, which stimulates the release of TEVs. Latent membrane protein 1 (LMP1) encapsulated by EVs promotes tumour growth in EBV-associated nasopharyngeal carcinomas [156] gastric cancer. In a liver metastasis model of colorectal cancer, these extracellular vesicles are absorbed by the liver. On one hand, they construct a pre-metastatic niche through the LATS2-YAP-MMP7 axis to facilitate CRC implantation in the liver, controlling the adhesion axis in hepatic tissue. On the other hand, they activate immune-suppressive signals mediated by the CD30-TRAF2-NF-κB pathway to remodel the metastatic microenvironment [157]. Within the PMN, macrophages often adopt the tumor-promoting M2 phenotype, characterized by increased Arg-1 expression and dependence on mitochondrial oxidative metabolism [158]. Macrophages treated with TEVs exhibit activation of the NF-κB pathway. This pathway utilizes HIF-1α/GLUT-1 to transport more glucose into macrophages and employs NOS2/NO to inhibit mitochondrial oxidative phosphorylation, consequently upregulating PD-L1 expression. Meanwhile, suppressed mitochondrial oxidative phosphorylation leads to the production of abundant lactate by macrophages, which, in a feedback loop, activates the NF-κB pathway to drive PD-L1 expression [159].

As previously mentioned, it is paramount not to overlook the pivotal role of let-7 s within EVs concerning the reshaping of the TME. In breast cancer, the content of Let-7 s in secreted EVs can be inhibited by Link28B, a type of RNA-binding protein. This induction prompts neutrophil infiltration into lung tissue and N2 transformation, and as the tumor progresses, this trend becomes increasingly pronounced. Furthermore, N2-transformed neutrophils suppress T cell proliferation, activation, and Th1 differentiation. Neutrophils undergoing N2 transformation exhibit high PD-L1 expression, limiting the tumor-killing capabilities of CD8 T cells. Additionally, this downregulation results in the upregulation of CXCL, IL-10, and IL-28 levels in lung fibroblasts, neutrophils, and macrophages, respectively [160]. These alterations in distant organ microenvironments create a conducive soil—an immunosuppressive PMN—that facilitates tumor metastasis.

### Altered vascular permeability and angiogenesis

In both in *vivo* and in vitro experiments, research has demonstrated that EVs play a pivotal role in promoting angiogenesis and altering vascular permeability [161]. Among these mechanisms, sprouting stands out as a vital form of neoangiogenesis [162]. EVs rich in clathrin light chain A (CLTA) have been shown to stimulate endothelial cells, further enhancing their tube-forming and sprouting capabilities [163]. Tip cells, located at the leading edge of sprouting, respond to signals in the microenvironment, thus influencing the growth trajectory of nascent blood vessels [164]. Evidence suggests that EVs derived from colorectal cancer cells, specifically those carrying circTUBGCP4, promote tip cell formation, angiogenesis, and consequently, tumor metastasis [165].

Adjacent vascular endothelial cells are linked and solidify endothelial integrity through vascular endothelial cadherin (VE-Cad) at their extracellular domains [166]. EVs from mesenchymal CRC cells transport miR-27b-3p into vascular endothelial cells. Further investigation has revealed that miR-27b-3p exerts its effects by directly binding to the 3′UTR of VE-Cad and p120, leading to post-transcriptional suppression of VE-Cad/P120 protein. This disruption compromises endothelial junction integrity and increases vascular permeability [167].

### Stromal component remodelling

Ovarian cancer derived EVs exhibit a proclivity for reprogramming stromal fibroblasts to shape the pre-metastatic niche (PMN) by secreting cytokines that promote metastasis via alterations in Hippo/YAP1 signaling cascade [168]. Following treatment with EVs rich in Cav-1 derived from breast cancer (BC), the expression of extracellular matrix (ECM) component proteins such as emilin1, nidogen, TnC, and FN increases in lung fibroblasts. Additionally, Cav-1-containing EVs mitigate the consequences of the silenced TnC gene in lung fibroblasts. This underscores the potential of Cav-1 in BC-derived EVs to transport TnC into lung stromal cells, facilitating the deposition of extracellular matrix proteins and acting as signaling molecules to promote the formation of the PMN during BC lung metastasis [169]. Fibroblasts, as vital constituents of the stroma, respond to signals conveyed by TEVs by upregulating CCL1 expression, thereby inducing the polarization of local microenvironmental Tregs [170]. In specific metastatic microenvironments, TEVs also play pivotal roles. In a bone metastasis model, the contents of TEVs, including miR-21, directly bind to PDCD4, promoting the activation and differentiation of osteoclasts without affecting osteoblasts. Consequently, this enhances local bone tissue osteoclast activity, leading to increased bone loss [171].

### Remodeling lymph node microenvironment

**Fig. 3:**
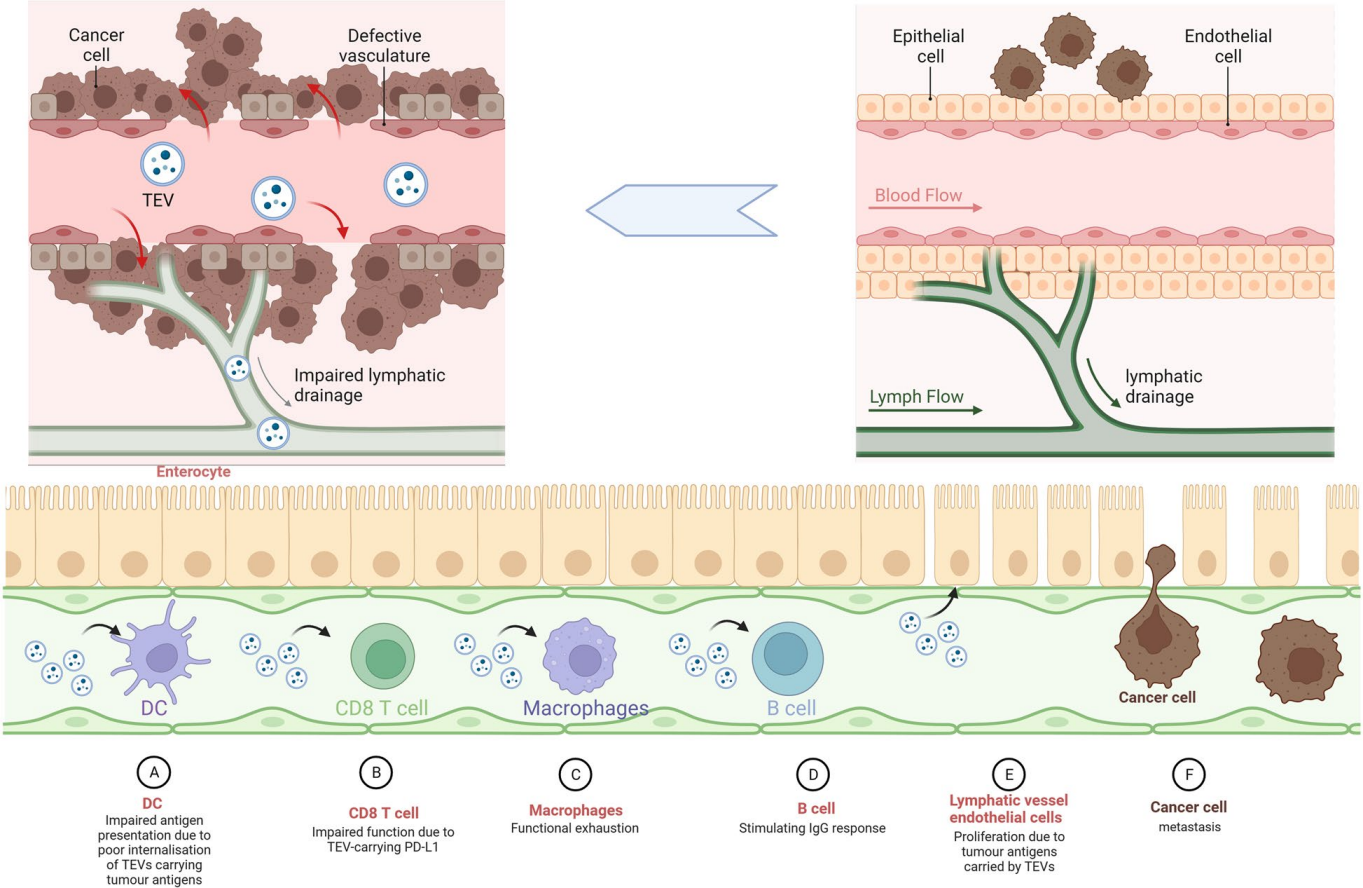
Extracellular vesicular remodelling of the lymphatic vascular microenvironment.** A** Impaired DC antigen presentation due to poor internalization of TEVs carrying tumor antigens. **B** TEV impairs and depletes TAM function. **C** TEV induces apoptosis of CD8T cells. **D** TEV activates B-cell anti-tumour IgG responses. **E** TEV induces lymphatic vessel endothelial cell proliferation, lymphatic vessel dilation and gap enlargement. **F** Metastasis of tumour cells from the dilated lymphatic vessel gap

Tumor-draining lymph nodes hold a pivotal role in the progression of cancer, serving as the initial destination of tumor metastasis and providing a significant prognostic indicator in various cancer types [172, 173]. These lymph nodes also have a crucial function in eliciting tumor-specific immunity and driving immunotherapeutic responses [174]. Tumor-secreted EVs play a regulatory role in the cytokine milieu within the lymph nodes, modulating the activity of immune cells. Specifically, they induce immunosuppression by upregulating pro-inflammatory cytokines such as IL-6 and TNF-α in lymph nodes, inhibiting tumor immune responses [175]. These EVs were taken up by macrophages and lymphocytes and decreased the cross-presentation of tumor antigens from DC cells, leading to a significant decrease in CD8 + T cell function in animal models [76] (Fig. 3a). Previous studies showed that lymphoepithelial cells in lymph nodes expressing PD-L1 triggered apoptosis of CD8 + T cells with tumor antigen specificity, thereby suppressing tumor immunity [176]. Another study found that TEVs, carrying PD-L1 reduced the number of CD8 + T cells infiltrating lymph nodes [55] (Fig. 3b).

In the early stages of tumorigenesis, macrophages residing within the lymph nodes act as a physical barrier to hinder the dissemination of TEVs. However, as the tumor advances, this macrophage barricade is dismantled, enabling the entry of TEVs carrying tumor-specific antigens deep into the cortical regions (Fig. 3c). Herein, TEVs stimulate B cells to generate pro-tumorigenic IgG, thereby promoting tumor progression [177] (Fig. 3d). Recently, VCAM-1 was found to play a crucial role in the transport of tumor-derived EVs to lymphatic vessels in a mouse model of B16F10 melanoma, leading to lymphatic vessel endothelial cell proliferation and LN remodeling, which ultimately resulted in lymphatic vessel expansion [76] (Fig. 3e). Furthermore, EVs derived from CRC were found to upregulate VEGFC expression in macrophages located in lymphatic vessels [178], which promoted tumor-associated lymphangiogenesis [179] (Fig. 3f).

## EVs as biomarkers in immunotherapy

**Fig. 4:**
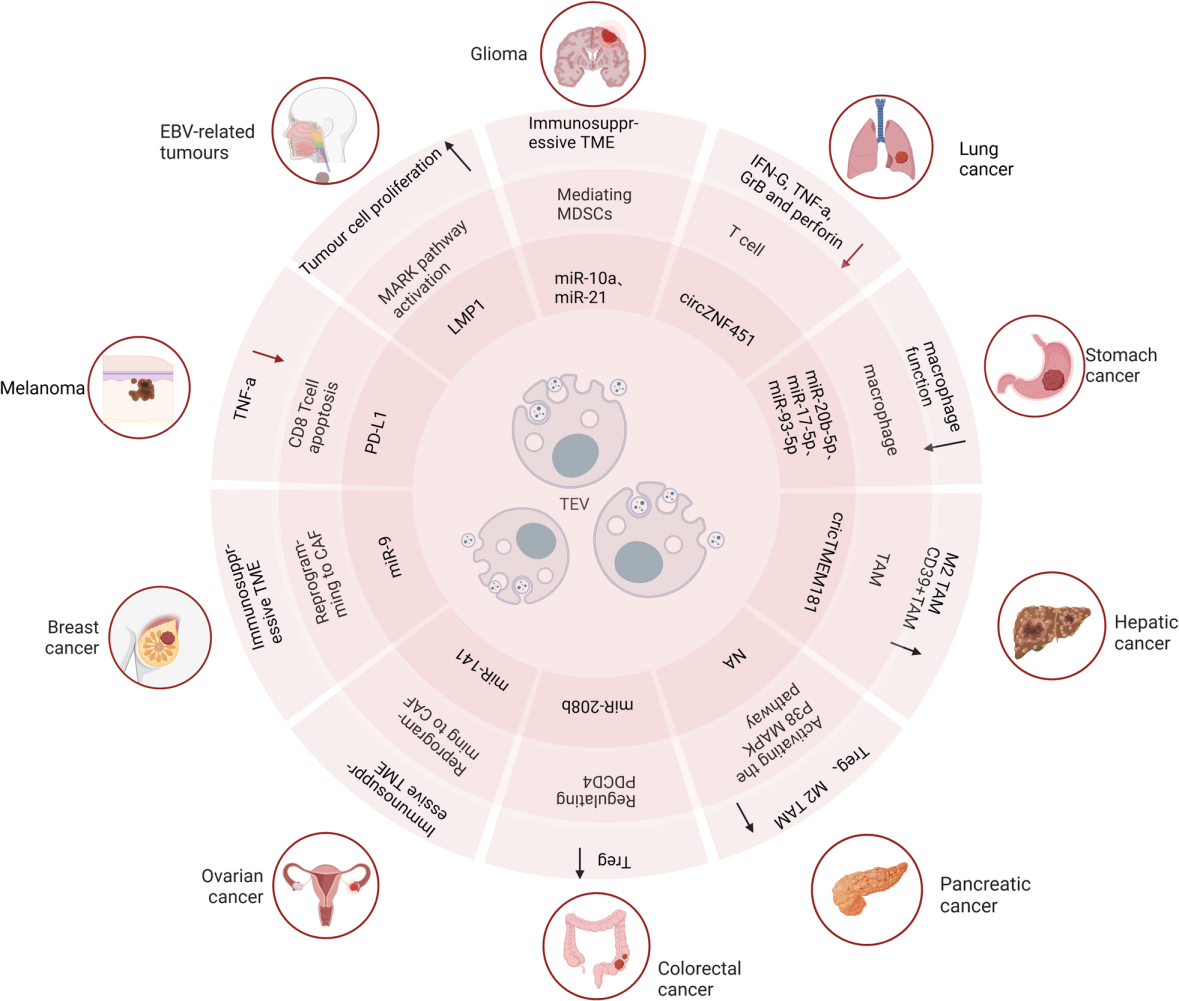
The effect of TEV on TME in different cancer types. TEV remodels TME in different cancer types, thus contributing to the shaping of a tumor-favorable microenvironment

Biomarkers in the realm of biology serve as indicators that reflect one or more physiological processes, pathogenic mechanisms, or responses to therapeutic interventions [180]. EVs play a pivotal role across a spectrum of malignancies (Fig. 4)). Tumor PD-L1 has already been employed as a predictive biomarker for clinical responses to anti-PD-1 therapies [181]. Intriguingly, PD-L1 encapsulated within EVs has garnered significant attention as an enhanced biomarker and has been validated across various cancer models [182, 183]. A groundbreaking development in this area involves the utilization of an immunogold biochip, enabling the quantification of RNA and proteins within individual EVs. This breakthrough substantially enhances the detection rate of EVs containing PD-1/PD-L1 mRNA [184].

EVs, at their core, constitute carriers of substances that facilitate intercellular communication signals. Certain cargo within EVs themselves can signify the body's immune state and its anti-tumor potency. Reports have linked high expression of LINC02096 (RIME) in plasma-derived EVs from cancer patients to reduced sensitivity to PD-1 monoclonal antibody treatment and adverse prognoses. Elevated RIME expression also correlates with diminished tumor-killing capabilities of CTLs [185].

A risk score reflecting the prognosis of cancer patients has been established based on EV proteins TNFRSF10B and ILF3 secreted by CAFs. Interestingly, no significant positive correlation has been discovered between immune cells and this risk score. However, when the TIDE score was employed to predict the outcomes and immune therapy responses of cancer patients [186], it was found that a high-risk score was significantly associated with poorer immune therapy (anti-PD1) efficacy. Survival analysis results further indicate that the high-risk group exhibits a less favorable prognosis after receiving immune therapy [187]. While certain EVs may not exhibit a clear association with immune therapy outcomes, they are closely linked to immune-related adverse events during such therapy. For instance, EV-ICOS and EV-IDO1 have proven to be robust predictors of immune-related adverse events in GC patients undergoing ICI therapy [188].

Although the available body of research is quite limited, some studies have begun to explore whether the contents of novel EVs can serve as biomarkers for predicting immune therapy responses. Recent research has unveiled that EVs harboring the urokinase-type plasminogen activator receptor (uPAR) may serve as novel biomarkers for intrinsic resistance to ICIs [189]. uPAR is intricately linked to tumor progression and metastasis [190]. Among patients treated with Pembrolizumab and nivolumab for immune therapy, non-responders exhibited baseline levels of uPAR + EVs similar to responders, albeit with significant differences in the source of these EVs. In contrast to responders, non-responder uPAR EVs, originating from melanoma cells, CD8 T cells, and DC cells, displayed significantly lower baseline levels. Significantly, uPAR EVs are correlated with patients' progression-free survival (PFS) and overall survival (OS), inversely associated with treatment outcomes [189]. Similarly, a comprehensive follow-up investigation discovered that the baseline high expression of TGF-β in EVs is associated with non-responsiveness to ICI therapy, as well as shorter PFS and OS. As a predictive biomarker, its efficacy surpasses that of circulating TGF-β levels and tissue PD-L1 content [183].

## Engineering EVs to reshape the TME to impact immunotherapy

**Fig. 5:**
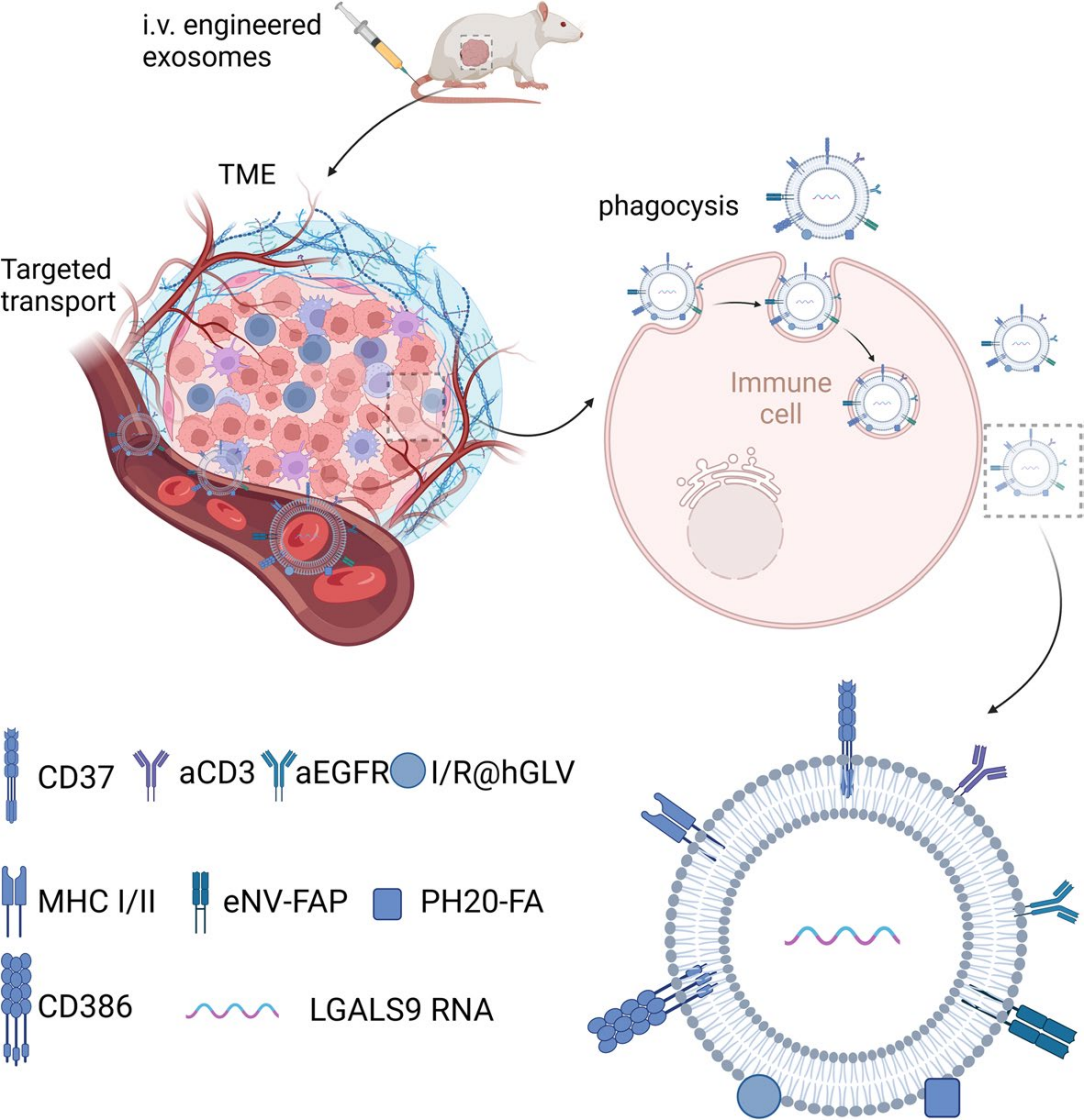
Schematic representation of engineered EVs implementing immunotherapy in a mouse model. After intravenous injection, engineered EVs carrying therapeutic molecules are targeted to the tumour site, followed by the release of immunotherapy-related drugs into the TME

Due to the pivotal role EVs play in tumor metastasis and therapy, researchers have embarked on the isolation and engineering of EVs to incorporate specific genes or proteins for employment in cancer treatment [191]. These bioengineered EVs are characterized by exceptional controllability, stability, and biocompatibility. They efficiently traverse vascular barriers and cellular membranes, enabling effective delivery of therapeutic agents into the intracellular milieu [192–194]. In recent years, engineered EVs have emerged prominently across various domains. They are manipulated via biotechnological approaches to harbor distinct cargoes, including therapeutic molecules or diagnostic markers, and are tailored for targeting specific cells or tissues. The versatility, stability, and biocompatibility of engineered EVs render them an appealing choice in biomedical applications, particularly in the realm of cancer research and treatment. Their potential to surmount biological barriers such as cell membranes and the blood–brain barrier holds exciting prospects for the development of novel therapies for a range of conditions, including oncology [195], cardiovascular diseases [196], tissue regeneration and repair [197], and neurological disorders [198], among others (Fig. 5).

Engineered EVs offer promising therapeutic opportunities for targeting and inhibiting tumor growth. The antitumor effects of EVs derived from M1 cells have been demonstrated, as they induced apoptosis in tumor cells. However, the underlying mechanism remains unclear [199]. Recently, CAR-T-derived EVs were found to inhibit the growth of triple-negative breast cancer (TNBC) cells expressing MSLn, possibly through the actions of perforin and granzyme B [200]. Researchers loaded target SiRNA into bone marrow MSC-derived EVs via electroporation and exploited the tumor homing ability of these EVs to block signaling communication between tumor cells and macrophages, which in turn inhibits TAM polarization and enhances immunotherapy in pancreatic ductal carcinoma [201].

Engineered EVs demonstrated their potential in improving immune cell function. A novel hGLV EV overexpressing CD47 has been found to enhance macrophage-mediated tumor cell phagocytosis by blocking CD47 signaling in tumor cells. Furthermore, the fusion of immune adjuvant with hGLV facilitated the maturation of DC cells, which in turn increased the infiltration of CD8 + and CD4 + T cells in tumors, resulting in improved anti-tumor immunity [202]. The recent rise of chimeric antigen receptor T (CAR T) cell therapy as a personalized, sequential cell therapy has gained much attention in the "war" against tumors [203, 204]. Taking cues from the personalized and sequential CAR T cell therapy, researchers edited DEXs to contain the CAR component of the MHC antigen peptide complex and the CD86 co-stimulatory molecule to activate T cells. Introduction of aCD3 and aEGFR further enhanced the binding of T cells to tumor tissue, leading to effective anti-tumor effects, particularly in solid tumors, and inhibition of tumor metastasis [205]. CAFs are also another important target for research. Previous studies showed that CAFs play a crucial role in the TME, and it is also an important mesenchymal target for immunotherapy of solid tumors [206]. To target CAFs, researchers developed eNV-FAP-containing EVs that trigger a strong specific cytotoxic effect on CTL immune responses leading to CAFs depletion. Furthermore, EVs-induced antitumor immunity also promoted iron death of tumor cells, which may be related to the release of IFN-γ and FAP + CAFs depletion by CTL [207].

Of course, engineered EVs also are used to make tumor vaccines. DCs, being essential for innate and adaptive immunity regulation in the TME, are the target of several DC-targeted vaccines in previous clinical trials to improve cancer immunotherapy. In previous clinical trials, several DC-targeted vaccines developed to improve cancer immunotherapy [208, 209]. The most recently developed EV vaccine against DC cells achieved good tumor suppression in a mouse model of breast cancer [210]. In addition, engineered EVs are loaded with drugs from other therapies, providing a combination therapy effect. Incorporation of PH20 with FA into EVs and injection into a mouse model revealed a reduction in infiltration of immunosuppressive cells such as Treg. It also reduced hyaluronidase-induced tumor cell metastasis and allowed enhanced delivery of chemotherapy through FA-modified tumor targeting [211]. Additionally, genetically engineered EVs fused with drug-loaded thermosensitive liposomes, named hGLV, have enabled photothermal therapy (PTT) under laser irradiation to kill tumors and tumor cell lysis, producing tumor-associated antigens, and promoting the maturation of dendritic cells to trigger a robust immune response with the help of co-encapsulated immune adjuvants [202]. Surface modification of LGALS-9-containing EVs with oxaliplatin (OXA) prodrugs also showed significant therapeutic benefits in cancer treatment when used in combination with chemotherapy [201].

It is worth mentioning that studies have reported the use of engineered EVPD1 to target PD-L1 in tumor cells to block the immunosuppressive effects of PD-L1 [212]. However, no engineered EVs are available for this pathway for the time being.

## Conclusion

In the intricate interplay between tumour and normal cells, EVs have emerged as key mediators of immune suppression and evasion. TEVs have been shown to subvert immune function, impairing the efficacy of immunotherapy. In contrast, normal cells, especially immune cells, also release EVs to counteract these effects, highlighting the crucial role of extracellular vesicular signals in regulating the immune response. The outcome of this struggle determines the fate of the tumour and the host. In the preclinical experimental phase, encompassing EVs including exosomes, their potential to impact immunotherapy in various cancers such as melanoma, lung cancer, liver cancer, colorectal cancer, breast cancer, and ovarian cancer has been observed (Table S1). Typically, TEVs harbor a plethora of immunosuppressive factors, such as PD-L1 and ncRNAs. However, it is noteworthy that interrupting EV release or taking intervention measures to impede the interaction between EV contents and receptor molecules can partially reverse this microenvironmental remodeling. Consequently, an augmentation in the efficacy of immunotherapy is evident. This immunotherapeutic sensitization is often reliant on the specific or signature molecules expressed by tumor cells or recipient cells. Deviating from this specificity may entail severe therapeutic side effects. As aforementioned, the body's immune system has persistently engaged in a relentless battle against tumors. Leveraging EVs generated by the immune system to combat cancer is another viable approach. Furthermore, the utilization of engineered EVs holds immense potential within the realm of tumor immunotherapy. These EVs possess distinctive targeting properties, allowing for the encapsulation of specific substances tailored to enhance therapeutic effects. Selecting appropriate EVs and cellular targets, standardizing engineered EVs, and developing reliable methods for EV extraction and quantification remain significant challenges. Regrettably, despite encouraging results in animal models, clinical translation remains in its infancy. The optimal therapeutic modalities, cargo selection, methods of EV cargo loading, and appropriate dosages for tumor treatment remain elusive. Perhaps drawing from the successful experiences of monoclonal antibody-based targeted therapies, proteins encapsulated within EVs may emerge as a predominant form of treatment in the future.

Nonetheless, the potential of EVs-mediated remodelling of the TME for enhancing immunotherapeutic sensitivity cannot be ignored. While more extensive research and validation are necessary, EVs offer unparalleled opportunities for revolutionizing tumour immunotherapy. As our understanding of EV biology deepens, we anticipate that these tiny vesicles will play an increasingly important role in the development of novel cancer treatments.

### Supplementary Information

**Additional file 1: Table S1.** EVs reshaping the TME and their impact on immunotherapy.

## Data Availability

Not applicable.
