# Supplementary material for: Extracellular vesicles remodel tumor environment for cancer immunotherapy
Source: Mol Cancer. 2023 Dec 13;22:203. doi: 10.1186/s12943-023-01898-5 (PMC10717809; doi:10.1186/s12943-023-01898-5)
Supplement: Supplementary file 1 — Additional file 1: Table S1. EVs reshaping the TME and their impact on immunotherapy. [file 12943_2023_1898_MOESM1_ESM.docx]

| **Origin of extracellular vesicles** | **Contents** | **Cancer types** | **Mechanisms** | **Relationship to immunotherapy** | **Ref.** |
| --- | --- | --- | --- | --- | --- |
| Melanoma cells | PD-L1 | Melanoma | PD-L1 induced T cell depletion | Direct attenuation of pembrolizumab treatment effect | [59] |
| Melanoma cells | Has-miR-3187-3p，has-miR-498，has-miR-122，has-miR149 and has-miR-181a /b | Melanoma | Regulation of TCR signalling and TNFα secretion | Indirectly related to immunotherapy, providing potential therapeutic targets | [62] |
| Non-small cell lung cancer | circUSP7 | Non-small cell lung cancer | Up-regulation of SHP2 expression inhibits CD8 T cell function and suppresses CD8 T cell secretion of IFN-γ, TNF-α, granzyme-B and perforin | Induction of anti-PD1 treatment resistance directly in a T cell-dependent form | [63] |
| Cancer cell | ICAM-1 and PD-L1 | Melanoma, Lung cancer and Colon cancer | ICAM-1-LFA-1-mediated adhesion between TEV and T cells induces PD-L1 to exert immunosuppression | Indirectly related to immunotherapy, providing inspiration for the development of immunotherapy vectors | [64] |
| Melanoma cells | tumorigen | Melanoma | Shuttling tumor antigens to LN LEC for cross-presentation on MHC-I leads to apoptosis induction in antigen-specific CD8 T cells | the elucidation of EV uptake or effector mechanisms in LEC may unveil novel targets for immunotherapy in melanoma | [65] |
| CD4 T cell | miR-25-3p、miR-155-5p、miR-215-5p and miR-375 | Melanoma | Stimulating CD8+ T cells without activating Tregs | Indirect impact immunotherapy ，providing a new direction for cancer immunotherapy T cell-mediated antitumor effects | [70] |
| Vδ2-T cell | FasL、TRAIL and immunostimulatory molecules | EBV-positive gastric cancer | Tumor cell killing and direct stimulation and activation of CD4 and CD8 T cells to enhance anti-tumor immunity | As a novel cell-free immunotherapy | [76] |
| Hepatocellular carcinoma | PD-L1 | Hepatocellular carcinoma | Upregulation of PD-L1 expression in TAM inhibits CD8 T cell function | Inhibition of PD-L1+TAM indirectly increases the efficacy of anti-PD-L1 immunotherapy | [78] |
| Hepatocellular carcinoma | circTMEM181 | Hepatocellular carcinoma | Up-regulation of CD39 expression in macrophages increases adenosine levels in TME and invalidates CD8 T cells | Activation of the ATP-adenosine pathway by EVs leads to poor response to anti-PD1 therapy directly | [84] |
| Lung adenocarcinoma | circZNF451 | lung adenocarcinoma | Induction of anti-inflammatory phenotype in macrophages and CD8 T cell exhaustion | Knockdown of ELF4 (circZNF451 downstream molecule) in macrophages indirectly rescues anti-PD1 immunotherapy effects | [87] |
| Hepatocellular carcinoma | circUHRF1 | Hepatocellular carcinoma | Inhibition of NK cell-derived IFN-γ and TNF-α secretion | circUHRF1 knockdown cells lead to increased sensitivity and overall survival to anti-PD1 therapy | [108] |
| Cancer cell | fatty acid | Breast cancer、Cervical cancer、Colon cancer、melanoma | DC immune dysfunction with reduced glycolysis | PPARα depletion directly affecting anti-PD-L1 antibody therapy | [98] |
| Engineered EVs | CD47、photothermal agent | Colon carcinoma | CD47 improving macrophage function, photothermal agent responding excellent photothermal therapy | Indirectly influencing immunotherapy as a combined therapeutic agent to improve therapeutic efficacy | [192] |
| Engineered EVs | MHC-antigen com-plexes 、CD86、anti-CD3 and anti-EGFR | Melanoma | CAR component for T cell activation, aCD3/aEGFR promotes T cell binding to cancer cells | Direct impact immunotherapy as a novel CAR-T therapy | [195] |
| Engineered EVs | fibroblast activation protein-α | Colon, Melanoma, Lung, and Breast Cancer | Recruitment of effector T cells and reduction of the proportion of immunosuppressive cells to suppress tumor growth | Directly related, with potential as a tumor vaccine | [197] |
